# Leveraging Real-World Data in Safety Signal Assessment

**DOI:** 10.1007/s43441-024-00682-x

**Published:** 2024-08-06

**Authors:** Vaishali Patadia, Katrin Manlik, Geoffrey Gipson, Jenna C. Willis, Ruth Namuyinga, Rachel McDermott, Anita Shaw, Mary K. Miller, Julius Asubonteng, Negar Golchin, Stephanie von Klot

**Affiliations:** 1 Amgen, One Amgen Center Drive, Thousand Oaks, CA 91320 USA; 2 Bayer AG, Berlin, Germany; 3 Global Medical Safety, Janssen, the Pharmaceutical Companies of Johnson & Johnson, Horsham, USA; 4 Roche Products Limited, Welwyn Garden City, UK; 5 Shionogi, Osaka, Japan; 6 Merck, Rahway, USA; 7 Genentech, A Member of the Roche Group, South San Francisco, USA; 8 Regeneron, Tarrytown, USA; 9 BMS, New York, USA; 10 Boehringer Ingelheim International GmbH, Ingelheim am Rhein, Germany

**Keywords:** Pharmacovigilance, Safety signal assessment, Real-world data, Rapid data analysis, Minimal protocol, TransCelerate

## Abstract

**Purpose:**

TransCelerate BioPharma surveyed its member biopharmaceutical companies to understand current practices and identify opportunities to complement safety signal assessment with rapid real-world data (RWD) analysis.

**Methods:**

A voluntary 30-question questionnaire regarding the use of RWD in safety signal assessment was disseminated to subject matter experts at all TransCelerate member companies in July 2022. Responses were blinded, aggregated, summarized, and presented.

**Results:**

Eighteen of 20 member companies provided responses to the questionnaire. Sixteen (89%) companies reported actively leveraging RWD in their signal assessment processes. Of 18 respondent companies, 8 (44%) routinely use rapid approaches to RWD analysis, 7 (39%) utilize rapid RWD analysis non-routinely or in a pilot setting, 2 (11%) are considering using rapid RWD analysis, and 1 (6%) has no plans to use rapid RWD analysis for their signal assessment. Most companies reported that RWD adds context to and improves quality of signal assessments. To conduct RWD analysis for signal assessment, 16 of 17 (94%) respondent companies utilize or plan to utilize internally available data, 8 (47%) utilize both internal and external data, and 3 (18%) utilize data networks. Respondents identified key challenges to rapidly performing RWD analyses, including data access/availability, time for analysis execution, and uncertainties regarding acceptance of minimal or non-protocolized approaches by health authorities.

**Conclusion:**

Biopharmaceutical companies reported that they see value in the use of rapid RWD analyses for complementing signal assessments. Future work is recommended to offer a framework and process for use of rapid use of RWD analyses in signal assessment.

**Supplementary Information:**

The online version contains supplementary material available at 10.1007/s43441-024-00682-x.

## Introduction

Health authorities continue to regulate the safety of a pharmaceutical product through the post-approval surveillance period, wherein regulatory bodies enforce pharmacovigilance, the monitoring of a drug’s potential risks, to ensure the ongoing safe use of the drug [1]. While the nuances vary by the governing authority, at its foundation, pharmacovigilance requires a signal management process to identify, evaluate, and appropriately respond to safety signals associated with the use of a drug to safeguard the public’s welfare [1, 2]. In the European Union (EU), for example, the signal management process consists of signal detection, signal validation, signal confirmation, signal analysis and prioritization, signal assessment, and recommendation for action [2].

An important central step of the signal management process is safety signal assessment, which involves evaluating a validated signal by considering all available evidence from different sources to confirm or refute the event as an adverse drug reaction [2]. While this step is required by health authorities, with guidance to be as comprehensive as possible regarding the sources of information, the specific methods and types of data used to fulfill this requirement are left open-ended [2]. As such, safety signal assessment typically relies on analysis of internal and external pharmacovigilance databases, clinical trial data, and scientific literature. However, these standard sources may be insufficient to conclude a signal assessment and often do not contain contextual longitudinal information detailing the patient’s treatment profile or the natural course of the event.

Conversely, real-world data (RWD) collected during routine clinical care can provide meaningful context to safety signal assessment and help safety evaluators to make appropriate decisions to protect public health [3]. RWD relate to patient health status, and/or delivery of health care and are derived from a variety of sources, including electronic health records and administrative claims data. Although RWD are more commonly used in pharmacoepidemiologic studies to generate real-world evidence (RWE) for the usage and potential benefits or risks of a drug, over the last decade, the use of RWD in pharmacovigilance has significantly increased to include safety signal assessment [4].

The use of RWD and the subsequent generation of RWE in pharmacovigilance typically involves the time-consuming and resource-intensive process of implementing a formal pharmacoepidemiologic study governed by a standard protocol. While such studies contribute robust evidence to the safety profile of a given medication, they are seldom feasible within tight regulatory timelines for signal assessment. For example, in the EU, signal assessment should be conducted within 3 months for important risks and within 6 months for non-important risks [2]. Thus, while the use of RWD in signal assessment offers added value, RWD are not widely employed in time-sensitive safety signal assessments across the biopharmaceutical industry. Incorporating RWE into a signal assessment necessitates expediting the availability of RWD analysis results to comply with regulatory timelines.

TransCelerate BioPharma is a non-profit organization with 20 biopharmaceutical member companies that aims to streamline and accelerate the research and development of new therapies around the world. To offer solutions addressing the resource and time constraints of traditional safety signal assessment, in January 2022, TransCelerate formed the Rapid Signal Assessment Using RWD (RSA-RWD) Initiative [5]. As part of this initiative, a questionnaire was provided to TransCelerate member companies to understand current practices and identify opportunities for use of rapid RWD analyses in safety signal assessment. Signal detection and signal validation were beyond the scope of this questionnaire.

## Materials and Methods

### Questionnaire

A questionnaire was designed to understand current practices and identify opportunities to use rapid RWD analysis for signal assessment in TransCelerate member companies, which include 20 multinational biopharmaceutical research and development organizations [6]. The questionnaire was sent to each member company’s representative (an RWD or pharmacovigilance expert identified by the member company) and distributed to their appropriate subject matter experts (SMEs). The company representative provided a single, consolidated response per company, which was accepted in the questionnaire tool (Sogolytics) between July 28, 2022 and August 16, 2022. The questionnaire is enclosed in the supplemental material (Online Appendix A).

The questionnaire was developed to understand how TransCelerate member companies define rapid signal assessment (RSA), explore the current state and future opportunities for RSA, and better understand the value, use of non-protocolized RSA, and regulatory requirements of RSA. As RSA is an emerging field, most concepts were exploratory. A draft questionnaire was tested by delegates from pharmacovigilance and RWE departments of member companies who were not part of the questionnaire development team. In addition, the questionnaire included an introductory letter outlining the background, objective, and definition of key terms used within the questionnaire. It included 30 questions on rapid RWD analysis usage, practices, benefits, and challenges. The questionnaire defined *rapid RWD analysis* as “an analysis of RWD which can be planned, conducted, documented, and interpreted within the required timeline for a safety signal assessment based on the member company’s internal timeline or external requirement from a health authority”. For the questionnaire, a rapid RWD analysis is not considered a formal observational or pharmacoepidemiologic study. These time frames for rapid analysis varied from “up to 1 week” (minimal) and “60–90 days” (maximum) in order to be considered rapid enough to meet organization’s or the regulatory agency’s timelines. Health authorities frequently request a response to signal assessments within 60–90 days. Hence, timelines “longer than 90 days” were not considered “rapid.” However, this response option was included in the questionnaire to ensure all possible time frames were covered.

In January 2023, a two-question follow-up was sent to member companies regarding the types of rapid RWD analyses performed for signal assessment and the type of protocol used (full, minimal, or none) for such analyses.

Questionnaire responses were voluntary and were blinded and aggregated by a third party. Response categories were mostly prespecified options, with some allowing for respondents to select multiple options or to specify “Other”. When appropriate, “Other” responses were recategorized to the available prespecified options. Some questions allowed for free text; these answers were reviewed and aggregated into higher level categories, where possible, for better interpretability. These categories were based on common themes identified during evaluation of questionnaire responses; if a new category was created, all previously analyzed responses were reassessed for that category.

### Analysis

Analysis categories were created based on the question, “Is your company currently leveraging RWD during safety signal assessment with a rapid RWD analysis approach which is not a formal observational study?” Member companies were categorized as either routine users of rapid RWD analysis if indicating “Yes, routinely used and integrated into business process” or as non-routine users if indicating “Yes, but not routinely (i.e. within a pilot setting)”, “Not yet, but concept is under consideration”, or “No”. Responses were summarized by frequencies and percentages based on non-missing responses. Results were presented as overall responses by respondent companies or stratified by current use of rapid RWD analysis for safety signal assessment (i.e., routine users versus non-routine users).

## Results

### Use of RWD in Signal Assessment

**Figure 1:**
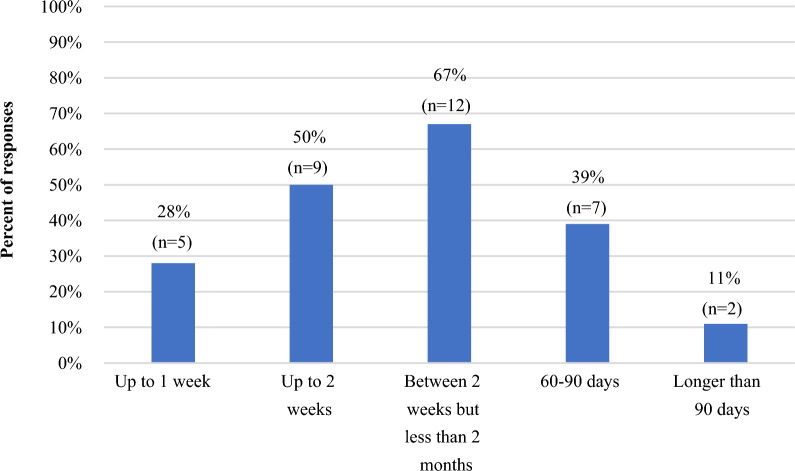
Acceptable time frame for delivery of rapid real-world data analysis in signal assessment reported by TransCelerate member companies (N = 18). Companies could select more than one response.

Eighteen of the 20 (90%) TransCelerate member companies responded to the 30-questions’ form, though not all companies answered each question. Of the 18 respondents, 16 (89%) indicated that they were actively leveraging RWD specifically for signal assessment, regardless of whether the approach was considered rapid or not. Some companies also specified using RWD in other areas of the signal management process, with 8/18 (44%) using RWD for signal detection and 7/18 (39%) for signal validation. When asked to provide acceptable time frames to fulfill company needs for the delivery of rapid RWD analysis for signal assessment, half (9/18) of the respondents specified 2 weeks or less (Fig. 1). Several companies indicated more than one time frame, with responses ranging from less than 1 week to more than 90 days.

### Current Use and Value of Rapid RWD Analysis

Out of 18 respondent companies, 8 (44%) reported routinely using a rapid approach to RWD analysis during safety signal assessment, 7 (39%) reported use of rapid RWD analysis, though not routinely, 2 (11%) indicated rapid RWD analysis as under consideration, and 1 (6%) reported no current plan to incorporate rapid RWD analysis. Most companies (14/18; 78%) reported that they plan to increase the use of rapid RWD analysis in signal assessment over the next 3 years.

Regarding the value of rapid RWD analysis, most companies indicated that RWD adds context to safety signal assessment (17/18; 94%) and enables decision-making (12/18; 67%). Companies also indicated that using rapid RWD analysis in signal assessment improves confidence in (16/17; 94%) and quality of the assessment (12/17; 71%) and reduces time to decision-making (12/17; 71%). Among companies currently using rapid RWD analyses, 7/8 (88%) routine users versus 5/9 (56%) non-routine users reported reduced decision-making time.

### Current and Planned Practices of Rapid RWD Analysis

When asked how rapid RWD analyses are or will be performed, nearly all respondent companies (16/17; 94%) reported conducting such analyses within their company using internal and/or in-licensed data. Nearly half of the companies (8/17; 47%) reported using external third-party analyses, and 3/17 (18%) reported leveraging data networks.

**Figure 2:**
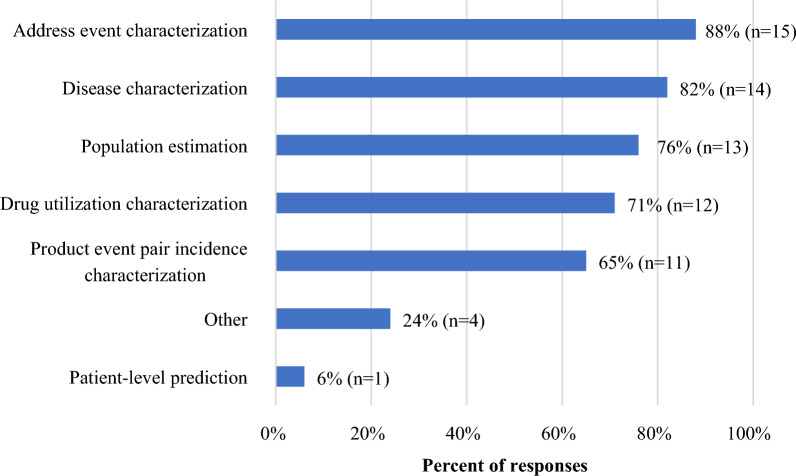
Types of planned and performed rapid real-world data analyses for signal assessment reported by TransCelerate member companies (N = 17). Companies could select more than one response.

Companies reported using or planning to use rapid RWD analysis across a variety of signal assessments, including for specific adverse events or outcomes, therapeutic areas or indications, populations, geographic regions, and signal priorities. The types of rapid RWD analyses planned or performed include both descriptive (characterizations of adverse events, disease, or drug utilization) and comparative analyses (population estimation, product event pair characterization, patient level prediction) (Fig. 2). Stratification by usage revealed no notable differences in the types of analyses planned or performed between the routine and non-routine users (data not shown).

**Figure 3:**
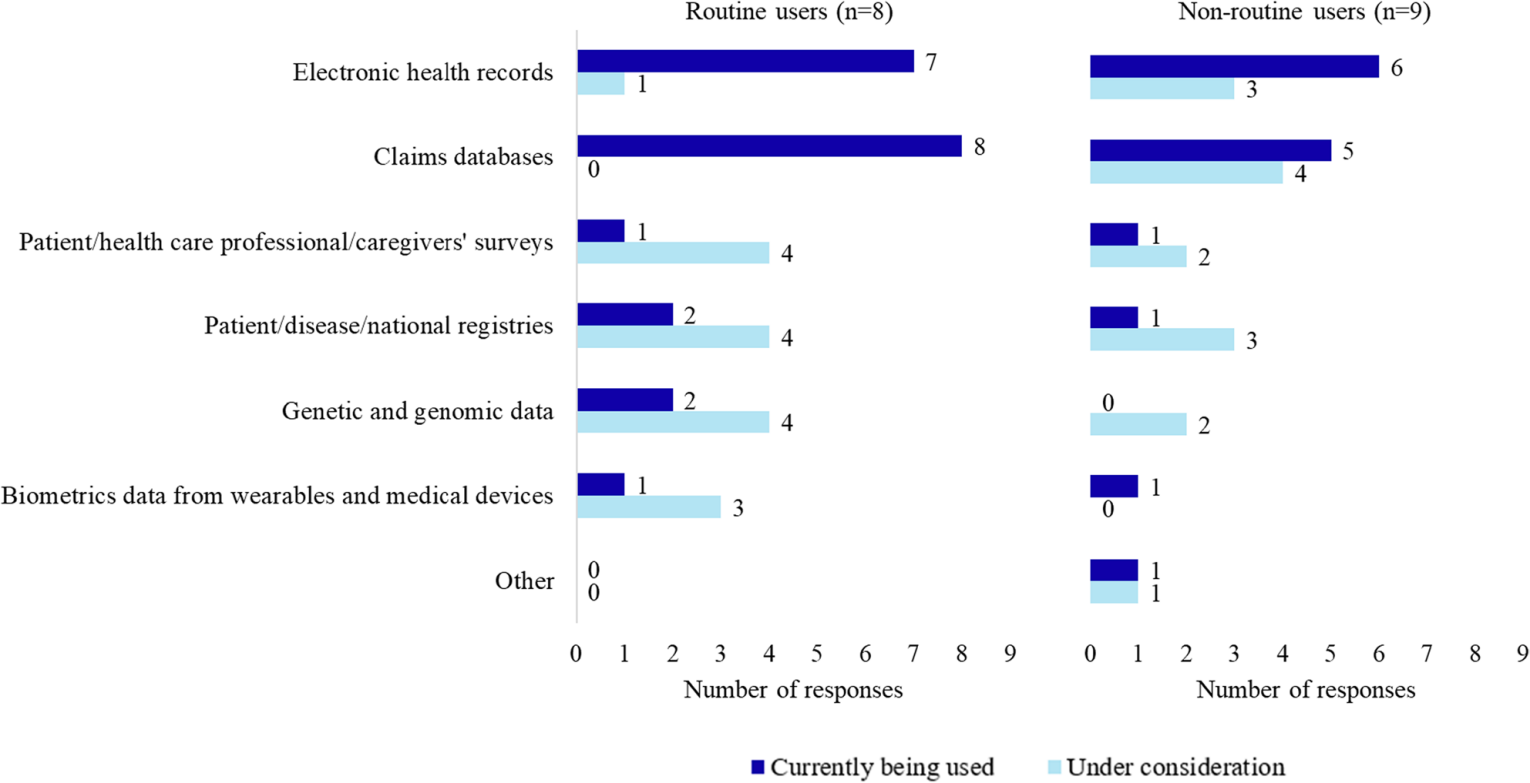
Real-world data (RWD) sources used or considered for use among routine (n = 8) and non-routine users (n = 9) of rapid RWD analyses in signal assessment. Companies could select more than one response.

Of 17 respondent companies, all use or are considering use of electronic health records and claims databases as sources for RWD (Fig. 3). Most routine users of rapid RWD analysis also indicated using or considering using claims databases, surveys, registries, or genetic and genomics data as RWD sources.

To implement rapid RWD analysis in signal assessment, 8/17 (47%) companies indicated using or planning to use a full protocol and statistical analysis plan, as with a formal observational study. Of 17 respondents, 14 (82%) reported use of a minimal protocol specifically focused on signal assessment, 4 (24%) specified other options, and 2 (12%) chose a non-protocolized approach, suggesting that protocol types are situational, with a minimal approach being preferred. Stratification by rapid RWD analysis usage revealed that 6/8 (75%) routine users versus 2/9 (22%) non-routine users considered a full protocol, and all 8 (100%) routine users versus 6/9 (67%) of the non-routine users considered a minimal protocol (data not shown). The follow-up questionnaire indicated that responding companies that use rapid RWD analyses (N = 12) tend to use a minimal protocol for analyses of background rates, disease and adverse events characterization, and population estimation. A full protocol is more often used for drug utilization, patient-level prediction, and product event characterization types of analyses.

To rapidly deliver RWD analysis, companies primarily streamlined or standardized efforts in execution and planning rather than protocol development and result documentation. Streamlined activities include analysis execution (13/17; 76%), acquiring access to relevant data sources (12/17; 71%), data source selection and feasibility (12/17; 71%), analysis parameterization (11/17; 65%), output development and visualizations (11/17; 65%), and establishment of relevant phenotype/code lists (9/17; 53%).

Among 17 respondents, 14 (82%) submitted results of rapid RWD analyses for signal assessment to a health authority. All 14 (100%) of these companies submitted to the European Medicines Agency (EMA) and 10/14 (71%) submitted to the United States Food and Drug Administration (FDA). Some companies indicated receiving methodological questions from health authorities regarding their rapid RWD analysis.

### Realized and Potential Benefits

Routine users estimate that half (median 50%) of signal assessments at their company use rapid RWD analysis, compared to a median 4% of assessments at companies with non-routine use (Table 1). Companies with routine use of rapid RWD analyses estimated a median 80% of signals assessed per year could benefit from such analyses, compared to a median 40% of signals at companies with non-routine use. The company-level gap between the frequency of actual and potentially valuable rapid RWD analyses for signal assessment was lower for routine users (median 10%) versus non-routine users (median 35%).

**Table 1:** Composite view of the sub-analyses of rapid signal assessment in the last year (stratified by current use of rapid RWD analysis).

	User	Measure					
		Median		1st Quartile, 3rd Quartile		Number of respondent companies	
		Non-routine [%]	Routine [%]	Non-routine [%]	Routine [%]	Non-routine	Routine
A	Percent of signal assessments that incorporated rapid RWD analysis (last year)	4	50	0, 10	30, 100	7	7
B	Estimated percent of signals assessed that could benefit from an additional rapid RWD analysis	40	80	25, 58	40, 100	8	7
C	Company-level difference of potential and actual percent of signal assessment incorporating rapid RWD analysis	35	10	16, 50	0, 30	6	7

### Challenges to Rapid RWD Analysis

Respondents were asked to identify the top three challenges to performing rapid RWD analysis in signal assessment. The most frequently reported challenge, identified by 8/18 (44%) companies, was time required for analysis execution. Two challenges were each ranked by 4 companies as their top challenge: availability or access to relevant RWD sources and uncertainty around acceptance of non-protocolized or minimally protocolized approaches by health authorities. Other reported challenges included establishing relevant phenotypes or code lists, establishing a protocol, and timeliness of available RWD data sources.

Though some companies indicated that these challenges have not yet been resolved, several specified various potential solutions. Notably, having analytical preparedness allowed companies to overcome many of the barriers to rapid RWD analysis. In addition, to address the time required for analysis execution, companies reported allocating appropriate resources, piloting, learning while growing, and developing internal expertise. Non-access to or unavailability of relevant RWD sources was addressed by active collaboration with data partners and investment in new data sources. To establish relevant phenotypes or code lists, companies required internal company collaboration, internal expertise, and process standardization. To address uncertainties around acceptance of the protocol approach by health authorities, some companies specified seeking feedback from the respective health authority. Process standardization was one approach to reducing time required for documentation.

## Discussion

Results from a questionnaire received from 18 biopharmaceutical member companies demonstrate a high level of interest in using rapid RWD analysis for safety signal assessment, with 89% of respondent companies using RWD in signal assessment. Acceptable time frames for rapid analyses ranged from less than 1 week to 90 days or more, suggesting that definitions of rapid analyses vary by company and situation. Users of RWD analysis reported that RWD add context to and increase confidence in signal assessments, and that RWD analyses reduce time to decision-making when done rapidly and properly. Rapid RWD analyses can bring awareness to potential safety issues sooner which, in turn, promotes patient safety and public health. Routine users indicated that most signal assessments could benefit from rapid RWD analysis while non-routine users showed a greater unmet need for rapid analysis.

Though member companies indicated there is both realized and unrealized value in RWD for signal assessment, questionnaire findings underscored several important barriers to performing these analyses rapidly to meet regulatory timelines. Challenges span the entire process of signal assessment, from problem definition, analysis planning, and execution to delivery and communication of results. In addition, there is uncertainty regarding the appropriateness and acceptability by health authorities of time-saving approaches to rapid RWD analyses, such as minimal or non-protocolized analysis, as there is limited guidance for companies in this area. While regulatory bodies have defined the use of RWD in the context of pharmacoepidemiologic studies to address safety questions, its rapid use in signal assessment has not been formalized.

A key challenge identified by member companies is the lack of clear expectations from health authorities regarding protocols for rapid RWD analysis in signal assessment, and whether a traditional protocol is required. Most respondent companies already used or planned to use a minimal protocol or no protocol to conduct rapid RWD analysis in signal assessment. A condensed protocol or analysis template with pre-defined minimal standards endorsed by health authorities would expedite the signal assessment process. Additionally, minimal endorsed standards for such protocols could potentially be incorporated into a common template or guidance document offering parameters for waiving the requirement of a formal protocol.

The challenges highlighted by this questionnaire also suggest a need for a clear descriptive framework for conducting rapid RWD analyses in signal assessment. To be useful, the framework should define roles and requirements for each step of the signal assessment process, including problem definition, data requirements, analytic planning, execution, and communication of results. For each step, the framework should outline the required documentation and criteria for moving forward and describe the differences and similarities between traditional RWD observational studies and rapid RWD analysis. The framework should also describe criteria for assessing the sufficiency of the rapid analysis to achieve the desired outcome.

Overall, the use of RWD in pharmacovigilance has significantly increased over the last decade. Despite this shift, fewer than 20% of respondent companies leverage existing data networks for rapid RWD analysis. Increasing the use of de-identified databases, which negate the need for informed consent, are essential to rapidly using RWD [7]. However, respondent companies listed identification and procurement of appropriate databases as key challenges to rapid RWD use. This suggests companies may be unfamiliar with the composition of different RWD sources and where/and how to access them. Patient attributes, including demographics, disease states, comorbidities, treatments, and care settings, are captured across a range of RWD sources. In addition, data elements may vary across regions, resulting in heterogeneous data capture within RWD sources. After a database has been identified and accessed, competencies in medicine, epidemiology, statistics, and health informatics may be required to decipher the data and derive meaningful conclusions. However, SMEs may not be readily available, or it may be logistically burdensome for companies to secure the required resources and personnel.

Fortunately, the increasing interest and investment in federated database solutions through collaborative networks may promote data access and availability. For example, the Observational Medical Outcomes Partnership (OMOP) [8] created a Common Data Model (CDM), a system to standardize observational health data. Ongoing network collaborations include the Observational Health Data Sciences and Informatics (OHDSI) [9] and the European Health Data & Evidence Network (EHDEN) [10]. Along with the utilization of CDMs, creation of pre-specified analytical tools, phenotype definitions, and validated software [11–13] would enable more rapid analysis of RWD. These networks involve partnerships between academia, regulatory, and pharmaceutical companies interested in developing resources to facilitate the use of RWD across multiple sources.

A highly promising wave of funding has been directed toward the use of RWD in regulatory decision-making from wider governmental bodies. Heads of Medicines Agency-EMA (HMA-EMA) joint Big Data Task Force has made use of the Horizon Europe (HORIZON) [14] funding for the Data Analysis and Real-World Interrogation Network (DARWIN EU®) [15] project. DARWIN EU® aims to support regulatory decision-making by establishing a catalogue of observational data sources, providing validated RWD, and carrying out non-interventional studies. While this project is not currently directed toward industry stakeholders, it is an important step forward in the potential use and regulatory acceptance of similar database networks. The Council for International Organizations of Medical Sciences (CIOMS) Working Group XIII (reference) recently published a consensus report on use of RWD and RWE in regulatory decision-making to support bridging the gap between efficacy information from clinical trials and effectiveness of medical products in real-world settings [16]. Collaboration with these networks provides important opportunities to leverage rapid RWD analyses for signal assessment.

While the questionnaire responses provide insight into current use and challenges of rapid RWD analysis for signal assessment, its limitations should be noted. This questionnaire sample was limited to multinational biopharmaceutical companies and their global functions. Thus, results may not represent activities in smaller pharmaceutical companies. Lastly, analyses were descriptive, with no formal statistical comparisons across companies or between routine and non-routine users of rapid RWD analysis.

## Conclusions

As part of TransCelerate Biopharma’s RSA-RWD Initiative, a perspective of 18 biopharmaceutical companies was provided to understand current practices and identify opportunities for use of rapid RWD analyses in safety signal assessment. Results from the questionnaire demonstrate that most companies are using or planning to use RWD in signal assessment. However, there are several challenges to conducting these analyses rapidly to meet regulatory timelines and standards, including time for analysis planning and execution, availability of RWD sources, and uncertainty regarding acceptance of minimal or non-protocolized approaches by health authorities. These challenges suggest the potential value of a clearly defined framework for conducting rapid RWD analysis to complement signal assessment. Opportunities to expedite RWD analysis include increasing database access and availability, developing templates or guidance documents for minimal protocols specific to signal assessment, and leveraging existing data networks.

## Supplementary Information

Below is the link to the electronic supplementary material.

Supplementary file1 (DOCX 128 KB)

## Data Availability

No datasets were generated or analysed during the current study.
