# Supplementary material for: Leveraging Real-World Data in Safety Signal Assessment
Source: Ther Innov Regul Sci. 2024 Aug 6;58(6):1062–70. doi: 10.1007/s43441-024-00682-x (PMC11530550; doi:10.1007/s43441-024-00682-x)
Supplement: Supplementary file 1 — Supplementary file1 (DOCX 128 KB) [file 43441_2024_682_MOESM1_ESM.docx]

| \| \| 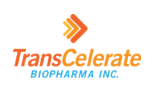 \| \| --- \| \| \| --- \| --- \| |
| --- | --- | --- |

| \| \| **TransCelerate Rapid Signal Assessment Using Real World Data Current State Survey** \| \| --- \| \|  \| \| \| --- \| --- \| --- \| \| \| \| \| **Introduction**  **Thank you for participating in this survey. TransCelerate has launched an initiative to gather current state information on the use of rapid Real World Data (RWD) analysis for safety signal assessment (i.e., signal evaluation). The goal of this TransCelerate Member Company survey is to understand the current state in the light of feasibility, speed, and regulatory requirements. Further, the survey intends to identify potential novel opportunities and approaches to complement signal assessment by rapid analysis of RWD to help overcome resource and time constraints and to potentially enhance patient safety and public health.**  **Please note that this survey is only focused on the safety signal assessment step in the Signal Management Process as seen in the figure below.**  **Signal Management Process (GVP Module IX)** \| \| --- \| \| \| --- \| --- \| \| \| --- \| --- \| --- \| \| \|  \| \| \| \| \| \| 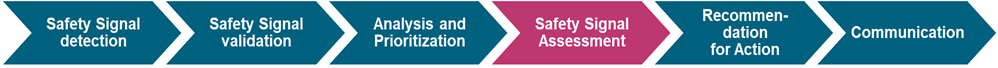 \| \| --- \| \|  \| \| \| --- \| --- \| --- \| \| \| --- \| --- \| --- \| --- \| \| \| **The survey results will be blinded and aggregated by a third party prior to being shared. Responding to any survey questions is entirely voluntary.**  **While your Member Company may have different definitions for the following, to ensure consistency in responses, it is important to leverage the following definitions in the context of this survey:  Survey Definitions:**  **Real World Data is defined per the FDA guidance and includes electronic health records, claims and bill activities, product and disease registries, patient-generated data, and data gathered from other sources that can inform on health status, such as mobile devices. Spontaneous adverse event reporting databases are NOT considered RWD.**  **Formal Observational Study  follows general required steps for preparing, conducting and executing a non-interventional study. Such studies require: 1.) a protocol which goes through formal review and approval phases and 2.) formal activities and planning for the execution and data analysis, preparation of study report, and regulatory reporting. Formal observational studies will typically address a safety concern and likely be listed as an additional PV activity with a defined due date.**  **Rapid RWD Analysis is defined as an analysis in RWD which can be planned, conducted, documented and interpreted within the required timeline for a safety assessment based on the Member Company’s internal timeline or external requirement from Health Authority. For example, rapid signal assessment would occur in the timelines to respond to signal assessment questions from Health Authorities or questions for routine safety surveillance reports (e.g., PSUR). However, if the evaluation was delayed until the next annual report, that would NOT be considered rapid. The exact definition of rapid may vary from company to company or safety topic to safety topic. In this survey we mean to characterize the range of durations associated with the concept of rapid.**  **Signal Assessment is defined as “The process of further evaluating a validated signal taking into account all available evidence, to determine whether there are new risks causally associated with the active substance or medicinal product or whether known risks have changed. This review may include nonclinical and clinical data and should be as comprehensive as possible regarding the sources of information.” (GVP Module IX). Signal Assessment begins AFTER the potential signal has been detected, validated, and prioritized. For example, the step of signal detection is out of scope for this survey.** \| \| --- \| \| \| \| --- \| --- \| --- \| --- \| --- \| --- \| --- \| \| \|  \| \| \| \| \| **1.** \| **Please indicate your TransCelerate Member Company. (Note: the results to this question will not be shared with anyone other than the third party aggregating the results)**  **(Select one)** \| \| --- \| --- \| \| \| --- \| --- \| --- \| \| \|  \| 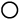 \| AbbVie \|  \| \| --- \| --- \| --- \| --- \| \|  \| 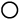 \| Amgen \|  \| \|  \| 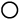 \| Astellas \|  \| \|  \| 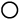 \| AstraZeneca \|  \| \|  \| 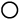 \| Bayer \|  \| \|  \| 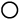 \| Boehringer Ingelheim \|  \| \|  \| 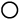 \| Bristol-Myers Squibb \|  \| \|  \| 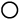 \| EMD Serono/Merck KGaA \|  \| \|  \| 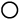 \| GlaxoSmithKline \|  \| \|  \| 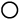 \| Johnson & Johnson \|  \| \|  \| 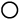 \| Eli Lilly \|  \| \|  \| 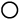 \| MSD/Merck & Co. \|  \| \|  \| 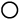 \| Novartis \|  \| \|  \| 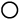 \| Novo Nordisk \|  \| \|  \| 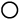 \| Pfizer \|  \| \|  \| 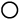 \| Regeneron \|  \| \|  \| 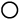 \| Roche \|  \| \|  \| 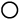 \| Sanofi \|  \| \|  \| 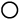 \| Shionogi \|  \| \|  \| 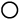 \| UCB \|  \| \| \| \| --- \| --- \| --- \| --- \| --- \| --- \| --- \| --- \| --- \| --- \| --- \| --- \| --- \| --- \| --- \| --- \| --- \| --- \| --- \| --- \| --- \| --- \| --- \| --- \| --- \| --- \| --- \| --- \| --- \| --- \| --- \| --- \| --- \| --- \| --- \| --- \| --- \| --- \| --- \| --- \| --- \| --- \| --- \| --- \| --- \| --- \| --- \| --- \| --- \| --- \| --- \| --- \| --- \| --- \| --- \| --- \| --- \| --- \| --- \| --- \| --- \| --- \| --- \| --- \| --- \| --- \| --- \| --- \| --- \| --- \| --- \| --- \| --- \| --- \| --- \| --- \| --- \| --- \| --- \| --- \| --- \| --- \| --- \| --- \| --- \| \| \|  \| \| \| \| \| **2.** \| **While the intention of this survey is on the signal assessment step in the Safety Signal Management Process outlined in GVP Module IX, please answer this question for all steps in the Signal Management Process (GVP module IX).**  **For which steps of the PV Signal Management process is your company actively leveraging RWD?   (Check all that apply)** \| \| --- \| --- \| \| \| --- \| --- \| --- \| \| \|  \| 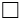 \| Signal Detection \|  \| \| --- \| --- \| --- \| --- \| \|  \| 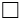 \| Signal Validation \|  \| \|  \| 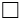 \| Analysis & Prioritization \|  \| \|  \| 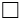 \| Signal Assessment \|  \| \|  \| 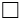 \| Recommendation for Action \|  \| \|  \| 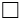 \| Communication \|  \| \|  \| 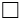 \| None of these steps \|  \| \|  \| 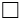 \| Other (Please specify)  ______________ \|  \| \| \| \| --- \| --- \| --- \| --- \| --- \| --- \| --- \| --- \| --- \| --- \| --- \| --- \| --- \| --- \| --- \| --- \| --- \| --- \| --- \| --- \| --- \| --- \| --- \| --- \| --- \| --- \| --- \| --- \| --- \| --- \| --- \| --- \| --- \| --- \| --- \| --- \| --- \| \| \|  \| \| \| \| \| **3.** \| **For what type of signal assessments has your company used, or is considering using rapid RWD data analysis?**  **(Check all that apply)** \| \| --- \| --- \| \| \| --- \| --- \| --- \| \| \|  \| 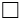 \| Specific therapeutic areas/indications (includes rare disease and long-latency diseases) \|  \| \| --- \| --- \| --- \| --- \| \|  \| 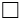 \| Specific geographical region \|  \| \|  \| 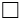 \| Specific populations \|  \| \|  \| 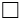 \| Specific priority of signals \|  \| \|  \| 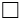 \| Specific adverse events or outcomes \|  \| \|  \| 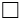 \| None \|  \| \|  \| 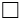 \| Other (Please specify)  ______________ \|  \| \| \| \| --- \| --- \| --- \| --- \| --- \| --- \| --- \| --- \| --- \| --- \| --- \| --- \| --- \| --- \| --- \| --- \| --- \| --- \| --- \| --- \| --- \| --- \| --- \| --- \| --- \| --- \| --- \| --- \| --- \| --- \| --- \| --- \| --- \| \| \|  \| \| \| \| \| **NOTE :** Answer the below question only if answer to Q#3 is Specific therapeutic areas/indications (includes rare disease and long-latency diseases) \| \| --- \|      \| **4.** \| **You indicated that your company uses or is considering using rapid RWD data analysis for a specific therapeutic area/ indication. Please specify the relevant therapeutic areas/ indications.** \| \| --- \| --- \| \| \| --- \| --- \| --- \| --- \| \|  \| \| \| --- \| --- \| --- \| --- \| --- \| --- \| \| \|  \| \| \| \| \| **5.** \| **From your company’s perspective, what does RWD analysis add to safety signal assessment? (Check all that apply)** \| \| --- \| --- \| \| \| --- \| --- \| --- \| \| \|  \| 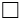 \| Context \|  \| \| --- \| --- \| --- \| --- \| \|  \| 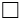 \| Confirmation \|  \| \|  \| 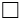 \| Enables decision making \|  \| \|  \| 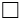 \| Other (Please specify)  ______________ \|  \| \| \| \| --- \| --- \| --- \| --- \| --- \| --- \| --- \| --- \| --- \| --- \| --- \| --- \| --- \| --- \| --- \| --- \| --- \| --- \| --- \| --- \| --- \| \| \|  \| \| \| \| \| **6.** \| **What are acceptable timeframes to fulfill your company’s needs regarding the delivery of rapid RWD analysis for signal assessment?  *(Please refer to the survey definition of Rapid RWD Analysis found in the introduction to answer this question)***  **(Check all that apply)** \| \| --- \| --- \| \| \| --- \| --- \| --- \| \| \|  \| 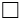 \| Up to 1 week \|  \| \| --- \| --- \| --- \| --- \| \|  \| 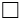 \| Up to 2 weeks \|  \| \|  \| 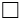 \| Between 2 weeks but less than 2 months \|  \| \|  \| 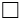 \| 60-90 days \|  \| \|  \| 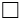 \| Longer than 90 days \|  \| \| \| \| --- \| --- \| --- \| --- \| --- \| --- \| --- \| --- \| --- \| --- \| --- \| --- \| --- \| --- \| --- \| --- \| --- \| --- \| --- \| --- \| --- \| --- \| --- \| --- \| --- \| \| \|  \| \| \| \| \| **7.** \| **Is your company currently leveraging RWD during safety signal assessment with a rapid RWD analysis approach which is not a formal observational study (please refer to the survey definitions)? (Select one)** \| \| --- \| --- \| \| \| --- \| --- \| --- \| \| \|  \| 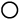 \| Yes, routinely used and integrated into business process \|  \| \| --- \| --- \| --- \| --- \| \|  \| 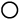 \| Yes, but not routinely (e.g., within in a pilot setting) \|  \| \|  \| 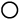 \| Not yet, but concept is under consideration \|  \| \|  \| 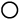 \| No \|  \| \| \| \| --- \| --- \| --- \| --- \| --- \| --- \| --- \| --- \| --- \| --- \| --- \| --- \| --- \| --- \| --- \| --- \| --- \| --- \| --- \| --- \| --- \| \| \|  \| \| \| \| **NOTE :** Answer the below question only if answer to Q#7 is Yes, routinely used and integrated into business process OR Yes, but not routinely (e.g., within in a pilot setting) OR Not yet, but concept is under consideration \| \| --- \| \| \| --- \| --- \| \| \| \| \| **8.** \| **Which types of RWD has your company used or are under consideration for future use within rapid RWD analyses in the context of signal assessment?** \| \| --- \| --- \| \| \| --- \| --- \| --- \| \| \| \| \| \| \| \| \| \| \| \| \| \| \| \| \| \| \| \| \| \| \| --- \| --- \| --- \| --- \| --- \| --- \| --- \| --- \| --- \| --- \| --- \| --- \| --- \| --- \| --- \| --- \| --- \| --- \| --- \| --- \| --- \| --- \| --- \| --- \| \|  \| **Currently Being Used** \| **Under Consideration** \|  \|  \|  \|  \|  \|  \|  \|  \|  \|  \|  \|  \|  \|  \|  \|  \|  \|  \| \| \|  \| (a) \| Electronic Health Records (EHR) \| \| --- \| --- \| --- \| \| \| 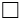 \|  \| \| --- \| --- \| \| \| 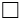 \|  \| \| --- \| --- \| \|  \|  \|  \|  \|  \|  \|  \|  \|  \|  \|  \|  \|  \|  \|  \|  \|  \|  \| \| \|  \| (b) \| Claims databases \| \| --- \| --- \| --- \| \| \| 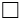 \|  \| \| --- \| --- \| \| \| 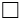 \|  \| \| --- \| --- \| \|  \|  \|  \|  \|  \|  \|  \|  \|  \|  \|  \|  \|  \|  \|  \|  \|  \|  \| \| \|  \| (c) \| Patient/Health Care Professional/caregivers' surveys \| \| --- \| --- \| --- \| \| \| 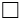 \|  \| \| --- \| --- \| \| \| 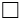 \|  \| \| --- \| --- \| \|  \|  \|  \|  \|  \|  \|  \|  \|  \|  \|  \|  \|  \|  \|  \|  \|  \|  \| \| \|  \| (d) \| Patient/disease/national registries \| \| --- \| --- \| --- \| \| \| 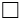 \|  \| \| --- \| --- \| \| \| 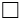 \|  \| \| --- \| --- \| \|  \|  \|  \|  \|  \|  \|  \|  \|  \|  \|  \|  \|  \|  \|  \|  \|  \|  \| \| \|  \| (e) \| Genetic and genomics data \| \| --- \| --- \| --- \| \| \| 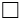 \|  \| \| --- \| --- \| \| \| 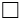 \|  \| \| --- \| --- \| \|  \|  \|  \|  \|  \|  \|  \|  \|  \|  \|  \|  \|  \|  \|  \|  \|  \|  \| \| \|  \| (f) \| Biometrics data from wearables and medical devices \| \| --- \| --- \| --- \| \| \| 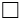 \|  \| \| --- \| --- \| \| \| 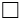 \|  \| \| --- \| --- \| \|  \|  \|  \|  \|  \|  \|  \|  \|  \|  \|  \|  \|  \|  \|  \|  \|  \|  \| \| \|  \| (g) \| Other (please specify) \| \| --- \| --- \| --- \| \| \| 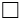 \|  \| \| --- \| --- \| \| \| 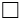 \|  \| \| --- \| --- \| \|  \|  \|  \|  \|  \|  \|  \|  \|  \|  \|  \|  \|  \|  \|  \|  \|  \|  \| \| \| \|  \| \| \| \| \| **NOTE :** Answer the below question only if answer to Q#7 is Yes, routinely used and integrated into business process OR Yes, but not routinely (e.g., within in a pilot setting) OR Not yet, but concept is under consideration \| \| --- \|      \| **9.** \| **Please indicate how rapid RWD analyses are or will be performed at your company.**  **(Check all that apply)** \| \| --- \| --- \| \| \| --- \| --- \| --- \| --- \| \| \|  \| 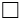 \| Internal analysis (e.g., analysis of company or in-licensed RWD data source) \|  \| \| --- \| --- \| --- \| --- \| \|  \| 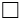 \| External third party analysis \|  \| \|  \| 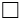 \| Leveraging data networks \|  \| \|  \| 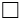 \| Other (Please specify)  ______________ \|  \| \| \| \| --- \| --- \| --- \| --- \| --- \| --- \| --- \| --- \| --- \| --- \| --- \| --- \| --- \| --- \| --- \| --- \| --- \| --- \| --- \| --- \| --- \| --- \| \| \|  \| \| \| \| \| **NOTE :** Answer the below question only if answer to Q#7 is Yes, routinely used and integrated into business process OR Yes, but not routinely (e.g., within in a pilot setting) OR Not yet, but concept is under consideration \| \| --- \|      \| **10.** \| **In your company’s experience, how does/ would leveraging rapid RWD analyses during signal assessment add value?**  **(Check all that apply)** \| \| --- \| --- \| \| \| --- \| --- \| --- \| --- \| \| \|  \| 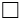 \| Reduced time to decision \|  \| \| --- \| --- \| --- \| --- \| \|  \| 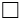 \| Improved quality \|  \| \|  \| 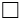 \| Improved confidence \|  \| \|  \| 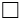 \| No value added \|  \| \|  \| 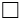 \| Other (Please specify)  ______________ \|  \| \| \| \| --- \| --- \| --- \| --- \| --- \| --- \| --- \| --- \| --- \| --- \| --- \| --- \| --- \| --- \| --- \| --- \| --- \| --- \| --- \| --- \| --- \| --- \| --- \| --- \| --- \| --- \| \| \|  \| \| \| \| \| **NOTE :** Answer the below question only if answer to Q#7 is Yes, routinely used and integrated into business process OR Yes, but not routinely (e.g., within in a pilot setting) OR Not yet, but concept is under consideration \| \| --- \|      \| **11.** \| **What types of analyses is your company performing or are planning to perform on RWD for signal assessment in a rapid approach?  (Check all that apply)** \| \| --- \| --- \| \| \| --- \| --- \| --- \| --- \| \| \|  \| 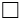 \| Disease characterization \|  \| \| --- \| --- \| --- \| --- \| \|  \| 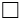 \| Drug utilization characterization \|  \| \|  \| 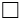 \| Adverse event characterization \|  \| \|  \| 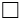 \| Product event pair incidence characterization \|  \| \|  \| 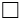 \| Population estimation \|  \| \|  \| 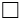 \| Patient level prediction \|  \| \|  \| 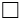 \| Other (Please specify)  ______________ \|  \| \| \| \| --- \| --- \| --- \| --- \| --- \| --- \| --- \| --- \| --- \| --- \| --- \| --- \| --- \| --- \| --- \| --- \| --- \| --- \| --- \| --- \| --- \| --- \| --- \| --- \| --- \| --- \| --- \| --- \| --- \| --- \| --- \| --- \| --- \| --- \| \| \|  \| \| \| \| \| **NOTE :** Answer the below question only if answer to Q#7 is Yes, routinely used and integrated into business process OR Yes, but not routinely (e.g., within in a pilot setting) OR Not yet, but concept is under consideration \| \| --- \|      \| **12.** \| **In the past 12 months, what percent of signal assessments at your company have incorporated rapid RWD analysis?**  **(Please provide a percent)** \| \| --- \| --- \| \| \| --- \| --- \| --- \| --- \| \|  \| \| \| --- \| --- \| --- \| --- \| --- \| --- \| \| \|  \| \| \| \| \| **13.** \| **Please estimate the % of signals assessed per year that could benefit from an additional rapid data analysis in RWD data.**  **(Please provide a percent)** \| \| --- \| --- \| \| \| --- \| --- \| --- \| \|  \| \| \| --- \| --- \| --- \| --- \| --- \| \| \|  \| \| \| \| \| **NOTE :** Answer the below question only if answer to Q#7 is Yes, routinely used and integrated into business process OR Yes, but not routinely (e.g., within in a pilot setting) OR Not yet, but concept is under consideration \| \| --- \|      \| **14.** \| **What type of protocol does your company use or are planning to use to enable rapid RWD analysis during signal assessment? (Check all that apply)** \| \| --- \| --- \| \| \| --- \| --- \| --- \| --- \| \| \|  \| 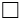 \| Full protocol and Statistical Analysis Plan, same or similar as for observational study \|  \| \| --- \| --- \| --- \| --- \| \|  \| 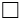 \| A minimal protocol (signal assessment plan/analysis plan/template) specifically focused on signal assessment \|  \| \|  \| 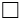 \| No protocol \|  \| \|  \| 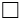 \| Other (Please specify)  ______________ \|  \| \| \| \| --- \| --- \| --- \| --- \| --- \| --- \| --- \| --- \| --- \| --- \| --- \| --- \| --- \| --- \| --- \| --- \| --- \| --- \| --- \| --- \| --- \| --- \| \| \|  \| \| \| \| \| **NOTE :** Answer the below question only if answer to Q#7 is Yes, routinely used and integrated into business process OR Yes, but not routinely (e.g., within in a pilot setting) OR Not yet, but concept is under consideration \| \| --- \|      \| **15.** \| **In which of the following areas has your company been able to streamline or standardize to rapidly deliver RWD analyses?**  **(Check all that apply)** \| \| --- \| --- \| \| \| --- \| --- \| --- \| --- \| \| \|  \| 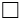 \| Planning: Getting access to relevant data sources \|  \| \| --- \| --- \| --- \| --- \| \|  \| 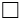 \| Planning: Data source selection and feasibility \|  \| \|  \| 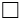 \| Planning: Development and approval of a protocol, if used (full, minimalized, template) \|  \| \|  \| 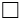 \| Planning: Establishing relevant phenotypes/code lists \|  \| \|  \| 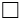 \| Execution: Analysis parametrization \|  \| \|  \| 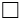 \| Execution: Analysis execution \|  \| \|  \| 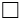 \| Execution: Output development and visualizations \|  \| \|  \| 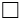 \| Execution: Results review and interpretation \|  \| \|  \| 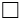 \| Result documentation and communication: Creation of final document/analysis output \|  \| \|  \| 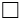 \| Result documentation and communication: Incorporating output into report/signal management documentation fit for external communication/submission \|  \| \|  \| 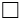 \| None \|  \| \|  \| 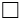 \| Other (Please specify)  ______________ \|  \| \| \| \| --- \| --- \| --- \| --- \| --- \| --- \| --- \| --- \| --- \| --- \| --- \| --- \| --- \| --- \| --- \| --- \| --- \| --- \| --- \| --- \| --- \| --- \| --- \| --- \| --- \| --- \| --- \| --- \| --- \| --- \| --- \| --- \| --- \| --- \| --- \| --- \| --- \| --- \| --- \| --- \| --- \| --- \| --- \| --- \| --- \| --- \| --- \| --- \| --- \| --- \| --- \| --- \| --- \| --- \| \| \|  \| \| \| \| \| **NOTE :** Answer the below question only if answer to Q#7 is Yes, routinely used and integrated into business process OR Yes, but not routinely (e.g., within in a pilot setting) OR Not yet, but concept is under consideration \| \| --- \|      \| **16.** \| **In what format does your company submit or plan to submit the results of the rapid RWD analysis to a Health Authority?**  **(Check all that apply)** \| \| --- \| --- \| \| \| --- \| --- \| --- \| --- \| \| \|  \| 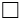 \| Creation of a stand-alone report \|  \| \| --- \| --- \| --- \| --- \| \|  \| 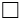 \| Integration into signal management document \|  \| \|  \| 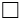 \| Integration into Health Authority Response document \|  \| \|  \| 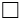 \| Other (Please specify)  ______________ \|  \| \| \| \| --- \| --- \| --- \| --- \| --- \| --- \| --- \| --- \| --- \| --- \| --- \| --- \| --- \| --- \| --- \| --- \| --- \| --- \| --- \| --- \| --- \| --- \| \| \|  \| \| \| \| \| **NOTE :** Answer the below question only if answer to Q#7 is Yes, routinely used and integrated into business process OR Yes, but not routinely (e.g., within in a pilot setting) OR Not yet, but concept is under consideration \| \| --- \|      \| **17.** \| **Has your company submitted the results of rapid RWD data analyses for signal assessment to a Health Authority? (Select one)** \| \| --- \| --- \| \| \| --- \| --- \| --- \| --- \| \| \|  \|  \| Yes \|  \| \| --- \| --- \| --- \| --- \| \|  \|  \| No \|  \| \| \| \| --- \| --- \| --- \| --- \| --- \| --- \| --- \| --- \| --- \| --- \| --- \| --- \| --- \| --- \| \| \|  \| \| \| \| \| **NOTE :** Answer the below question only if answer to Q#17 is Yes \| \| --- \|      \| **18.** \| **Please select the Health Authorities to which your company has submitted rapid RWD analysis for signal assessment. (Check all that apply)** \| \| --- \| --- \| \| \| --- \| --- \| --- \| --- \| \| \|  \|  \| FDA \|  \| \| --- \| --- \| --- \| --- \| \|  \|  \| EMA \|  \| \|  \|  \| MHRA \|  \| \|  \|  \| PMDA \|  \| \|  \|  \| Other (Please specify)  ______________ \|  \| \| \| \| --- \| --- \| --- \| --- \| --- \| --- \| --- \| --- \| --- \| --- \| --- \| --- \| --- \| --- \| --- \| --- \| --- \| --- \| --- \| --- \| --- \| --- \| --- \| --- \| --- \| --- \| \| \|  \| \| \| \| \| **NOTE :** Answer the below question only if answer to Q#17 is Yes \| \| --- \|      \| **19.** \| **What type of questions, concerns, or feedback did the Health Authority have? (Free text)** \| \| --- \| --- \| \| \| --- \| --- \| --- \| --- \| \|  \| \| \| --- \| --- \| --- \| --- \| --- \| --- \| \| \|  \| |
| --- | --- | --- | --- | --- | --- | --- | --- | --- | --- | --- | --- | --- | --- | --- | --- | --- | --- | --- | --- | --- | --- | --- | --- | --- | --- | --- | --- | --- | --- | --- | --- | --- | --- | --- | --- | --- | --- | --- | --- | --- | --- | --- | --- | --- | --- | --- | --- | --- | --- | --- | --- | --- | --- | --- | --- | --- | --- | --- | --- | --- | --- | --- | --- | --- | --- | --- | --- | --- | --- | --- | --- | --- | --- | --- | --- | --- | --- | --- | --- | --- | --- | --- | --- | --- | --- | --- | --- | --- | --- | --- | --- | --- | --- | --- | --- | --- | --- | --- | --- | --- | --- | --- | --- | --- | --- | --- | --- | --- | --- | --- | --- | --- | --- | --- | --- | --- | --- | --- | --- | --- | --- | --- | --- | --- | --- | --- | --- | --- | --- | --- | --- | --- | --- | --- | --- | --- | --- | --- | --- | --- | --- | --- | --- | --- | --- | --- | --- | --- | --- | --- | --- | --- | --- | --- | --- | --- | --- | --- | --- | --- | --- | --- | --- | --- | --- | --- | --- | --- | --- | --- | --- | --- | --- | --- | --- | --- | --- | --- | --- | --- | --- | --- | --- | --- | --- | --- | --- | --- | --- | --- | --- | --- | --- | --- | --- | --- | --- | --- | --- | --- | --- | --- | --- | --- | --- | --- | --- | --- | --- | --- | --- | --- | --- | --- | --- | --- | --- | --- | --- | --- | --- | --- | --- | --- | --- | --- | --- | --- | --- | --- | --- | --- | --- | --- | --- | --- | --- | --- | --- | --- | --- | --- | --- | --- | --- | --- | --- | --- | --- | --- | --- | --- | --- | --- | --- | --- | --- | --- | --- | --- | --- | --- | --- | --- | --- | --- | --- | --- | --- | --- | --- | --- | --- | --- | --- | --- | --- | --- | --- | --- | --- | --- | --- | --- | --- | --- | --- | --- | --- | --- | --- | --- | --- | --- | --- | --- | --- | --- | --- | --- | --- | --- | --- | --- | --- | --- | --- | --- | --- | --- | --- | --- | --- | --- | --- | --- | --- | --- | --- | --- | --- | --- | --- | --- | --- | --- | --- | --- | --- | --- | --- | --- | --- | --- | --- | --- | --- | --- | --- | --- | --- | --- | --- | --- | --- | --- | --- | --- | --- | --- | --- | --- | --- | --- | --- | --- | --- | --- | --- | --- | --- | --- | --- | --- | --- | --- | --- | --- | --- | --- | --- | --- | --- | --- | --- | --- | --- | --- | --- | --- | --- | --- | --- | --- | --- | --- | --- | --- | --- | --- | --- | --- | --- | --- | --- | --- | --- | --- | --- | --- | --- | --- | --- | --- | --- | --- | --- | --- | --- | --- | --- | --- | --- | --- | --- | --- | --- | --- | --- | --- | --- | --- | --- | --- | --- | --- | --- | --- | --- | --- | --- | --- | --- | --- | --- | --- | --- | --- | --- | --- | --- | --- | --- | --- | --- | --- | --- | --- | --- | --- | --- | --- | --- | --- | --- | --- | --- | --- | --- | --- | --- | --- | --- | --- | --- | --- | --- | --- | --- | --- | --- | --- | --- | --- | --- | --- | --- | --- | --- | --- | --- | --- | --- | --- | --- | --- | --- | --- | --- | --- | --- | --- | --- | --- | --- | --- | --- | --- | --- | --- | --- | --- | --- | --- | --- | --- | --- | --- | --- | --- | --- | --- | --- | --- | --- | --- | --- | --- | --- | --- | --- | --- | --- | --- | --- | --- | --- | --- | --- | --- | --- | --- | --- | --- | --- | --- | --- | --- | --- | --- | --- | --- | --- | --- | --- | --- | --- | --- | --- | --- | --- | --- | --- | --- | --- | --- | --- | --- | --- | --- | --- | --- | --- | --- | --- | --- | --- | --- | --- | --- | --- | --- | --- | --- | --- | --- | --- | --- | --- | --- | --- | --- | --- | --- | --- | --- | --- | --- | --- | --- | --- | --- | --- | --- | --- | --- | --- | --- | --- | --- | --- | --- | --- | --- | --- | --- | --- | --- | --- | --- | --- | --- | --- | --- | --- | --- | --- | --- | --- | --- | --- | --- | --- | --- | --- | --- | --- | --- | --- | --- | --- | --- | --- | --- | --- | --- | --- | --- | --- | --- | --- | --- | --- | --- | --- | --- | --- | --- | --- | --- | --- | --- | --- | --- | --- | --- | --- | --- | --- | --- | --- | --- | --- | --- | --- | --- | --- | --- | --- | --- | --- | --- | --- | --- | --- | --- | --- | --- | --- | --- | --- | --- | --- | --- | --- | --- | --- | --- | --- | --- | --- | --- | --- | --- | --- | --- | --- | --- | --- | --- | --- | --- | --- | --- | --- | --- | --- | --- | --- | --- | --- | --- | --- | --- | --- | --- | --- | --- | --- | --- | --- | --- | --- | --- | --- | --- | --- | --- | --- | --- | --- | --- | --- | --- | --- | --- | --- | --- | --- | --- | --- | --- | --- | --- | --- | --- | --- | --- | --- | --- | --- | --- | --- | --- | --- | --- | --- | --- | --- | --- | --- | --- | --- | --- |

| \|  \| \| --- \| \| \| \| \| **Instructions:  In the next set of questions, you will be asked to identify the top 3 challenges or barriers your company is currently experiencing from the list below when using rapid RWD analyses in signal assessment.  You will be asked this question 3 times to allow you to enter your top 3 challenges or barriers.  If you do not see all of your top 3 challenges in the list below, you may use "other", describing the challenge not in the list.  Selecting each challenge will then be followed by a question about the ways your company may be overcoming that challenge or barrier.    List of potential challenges or barriers from which you will select:**   - **Availability or access to relevant RWD data sources** - **Timeliness of available RWD data sources** - **Availability or access to suitable RWD analytic tools** - **Capabilities of available RWD analytic tools** - **Time required for data feasibility checks/data source selection** - **Establishing relevant phenotypes/code lists incl. mapping of codes between ontologies (e.g., MedDRA to ICD)** - **Establishing a protocol, if used (full, minimal, template)** - **Establishing pre-specified analyses** - **Time required for analysis execution (e.g., parametrization, execution, output development and visualizations, results review and interpretation)** - **Time required for result documentation (e.g., creation of final document/analysis output, incorporating output into report/signal management documentation fit for external communication/submission)** - **Uncertainty around acceptance of non-protocolized/minimal protocolized approach by health authorities** - **Uncertainty around report formats accepted by health authorities** - **Uncertainty around reporting compliance for Individual Case Safety Reports (ICSRs)** - **Other (Please specify)** \| \| --- \| \| \| --- \| --- \| \| \| --- \| --- \| --- \| \| \|  \| \| \| \| \| **20.** \| Please select your company’s top 3 challenges or barriers in order of importance (Selection 1 of 3)  **(Select one option)** \| \| --- \| --- \| \| \| --- \| --- \| --- \| \| \|  \|  \| Availability or access to relevant RWD data sources \|  \| \| --- \| --- \| --- \| --- \| \|  \|  \| Timeliness of available RWD data sources \|  \| \|  \|  \| Availability or access to suitable RWD analytic tools \|  \| \|  \|  \| Capabilities of available RWD analytic tools \|  \| \|  \|  \| Time required for data feasibility checks/data source selection \|  \| \|  \|  \| Establishing relevant phenotypes/code lists incl. mapping of codes between ontologies (e.g. MedDRA to ICD) \|  \| \|  \|  \| Establishing a protocol, if used (full, minimal, template) \|  \| \|  \|  \| Establishing pre-specified analyses \|  \| \|  \|  \| Time required for analysis execution \|  \| \|  \|  \| Time required for result documentation \|  \| \|  \|  \| Uncertainty around acceptance of non-protocolized/minimal protocolized approach by health authorities \|  \| \|  \|  \| Uncertainty around report formats accepted by health authorities \|  \| \|  \|  \| Uncertainty around reporting compliance for ICSRs \|  \| \|  \|  \| Other (Please specify)  __________ \|  \| \| \| \| --- \| --- \| --- \| --- \| --- \| --- \| --- \| --- \| --- \| --- \| --- \| --- \| --- \| --- \| --- \| --- \| --- \| --- \| --- \| --- \| --- \| --- \| --- \| --- \| --- \| --- \| --- \| --- \| --- \| --- \| --- \| --- \| --- \| --- \| --- \| --- \| --- \| --- \| --- \| --- \| --- \| --- \| --- \| --- \| --- \| --- \| --- \| --- \| --- \| --- \| --- \| --- \| --- \| --- \| --- \| --- \| --- \| --- \| --- \| --- \| --- \| \| \|  \| \| \| \| \| **21.** \| **How is your company overcoming this challenge or barrier?** \| \| --- \| --- \| \| \| --- \| --- \| --- \| \|  \| \| \| --- \| --- \| --- \| --- \| --- \| \| \|  \| \|  \| \|  \| \|  \| \|  \| \|  \| \|  \| \|  \| \|  \| \|  \| \|  \| \|  \| \|  \| \|  \| \|  \| \|  \| \| \|  \| \| --- \| \|  \| \| |
| --- | --- | --- | --- | --- | --- | --- | --- | --- | --- | --- | --- | --- | --- | --- | --- | --- | --- | --- | --- | --- | --- | --- | --- | --- | --- | --- | --- | --- | --- | --- | --- | --- | --- | --- | --- | --- | --- | --- | --- | --- | --- | --- | --- | --- | --- | --- | --- | --- | --- | --- | --- | --- | --- | --- | --- | --- | --- | --- | --- | --- | --- | --- | --- | --- | --- | --- | --- | --- | --- | --- | --- | --- | --- | --- | --- | --- | --- | --- | --- | --- | --- | --- | --- | --- | --- | --- | --- | --- | --- | --- | --- | --- | --- | --- |
|  |

| \|  \| \| --- \| \| \| \| \| **22.** \| Please select your company’s top 3 challenges or barriers in order of importance (Selection 2 of 3)    **(Select one option)** \| \| --- \| --- \| \| \| --- \| --- \| --- \| \| \|  \|  \| Availability or access to relevant RWD data sources \|  \| \| --- \| --- \| --- \| --- \| \|  \|  \| Timeliness of available RWD data sources \|  \| \|  \|  \| Availability or access to suitable RWD analytic tools \|  \| \|  \|  \| Capabilities of available RWD analytic tools \|  \| \|  \|  \| Time required for data feasibility checks/data source selection \|  \| \|  \|  \| Establishing relevant phenotypes/code lists incl. mapping of codes between ontologies (e.g. MedDRA to ICD) \|  \| \|  \|  \| Establishing a protocol, if used (full, minimal, template) \|  \| \|  \|  \| Establishing pre-specified analyses \|  \| \|  \|  \| Time required for analysis execution \|  \| \|  \|  \| Time required for result documentation \|  \| \|  \|  \| Uncertainty around acceptance of non-protocolized/minimal protocolized approach by health authorities \|  \| \|  \|  \| Uncertainty around report formats accepted by health authorities \|  \| \|  \|  \| Uncertainty around reporting compliance for ICSRs \|  \| \|  \|  \| Other (Please specify)  __________ \|  \| \| \| \| --- \| --- \| --- \| --- \| --- \| --- \| --- \| --- \| --- \| --- \| --- \| --- \| --- \| --- \| --- \| --- \| --- \| --- \| --- \| --- \| --- \| --- \| --- \| --- \| --- \| --- \| --- \| --- \| --- \| --- \| --- \| --- \| --- \| --- \| --- \| --- \| --- \| --- \| --- \| --- \| --- \| --- \| --- \| --- \| --- \| --- \| --- \| --- \| --- \| --- \| --- \| --- \| --- \| --- \| --- \| --- \| --- \| --- \| --- \| --- \| --- \| \| \| \| \| \| **23.** \| **How is your company overcoming this challenge or barrier?** \| \| --- \| --- \| \| \| --- \| --- \| --- \| \|  \| \| \| --- \| --- \| --- \| --- \| --- \| \| \|  \| \|  \| \| \| \| \| **24.** \| Please select your company’s top 3 challenges or barriers in order of importance (Selection 3 of 3)    **(Select one option)** \| \| --- \| --- \| \| \| --- \| --- \| --- \| \| \|  \|  \| Availability or access to relevant RWD data sources \|  \| \| --- \| --- \| --- \| --- \| \|  \|  \| Timeliness of available RWD data sources \|  \| \|  \|  \| Availability or access to suitable RWD analytic tools \|  \| \|  \|  \| Capabilities of available RWD analytic tools \|  \| \|  \|  \| Time required for data feasibility checks/data source selection \|  \| \|  \|  \| Establishing relevant phenotypes/code lists incl. mapping of codes between ontologies (e.g. MedDRA to ICD) \|  \| \|  \|  \| Establishing a protocol, if used (full, minimal, template) \|  \| \|  \|  \| Establishing pre-specified analyses \|  \| \|  \|  \| Time required for analysis execution \|  \| \|  \|  \| Time required for result documentation \|  \| \|  \|  \| Uncertainty around acceptance of non-protocolized/minimal protocolized approach by health authorities \|  \| \|  \|  \| Uncertainty around report formats accepted by health authorities \|  \| \|  \|  \| Uncertainty around reporting compliance for ICSRs \|  \| \|  \|  \| Other (Please specify)  __________ \|  \| \| \| \| --- \| --- \| --- \| --- \| --- \| --- \| --- \| --- \| --- \| --- \| --- \| --- \| --- \| --- \| --- \| --- \| --- \| --- \| --- \| --- \| --- \| --- \| --- \| --- \| --- \| --- \| --- \| --- \| --- \| --- \| --- \| --- \| --- \| --- \| --- \| --- \| --- \| --- \| --- \| --- \| --- \| --- \| --- \| --- \| --- \| --- \| --- \| --- \| --- \| --- \| --- \| --- \| --- \| --- \| --- \| --- \| --- \| --- \| --- \| --- \| --- \| \| \| \| \| \| **25.** \| **How is your company overcoming this challenge or barrier?** \| \| --- \| --- \| \| \| --- \| --- \| --- \| \|  \| \| \| --- \| --- \| --- \| --- \| --- \| \| \|  \| |
| --- | --- | --- | --- | --- | --- | --- | --- | --- | --- | --- | --- | --- | --- | --- | --- | --- | --- | --- | --- | --- | --- | --- | --- | --- | --- | --- | --- | --- | --- | --- | --- | --- | --- | --- | --- | --- | --- | --- | --- | --- | --- | --- | --- | --- | --- | --- | --- | --- | --- | --- | --- | --- | --- | --- | --- | --- | --- | --- | --- | --- | --- | --- | --- | --- | --- | --- | --- | --- | --- | --- | --- | --- | --- | --- | --- | --- | --- | --- | --- | --- | --- | --- | --- | --- | --- | --- | --- | --- | --- | --- | --- | --- | --- | --- | --- | --- | --- | --- | --- | --- | --- | --- | --- | --- | --- | --- | --- | --- | --- | --- | --- | --- | --- | --- | --- | --- | --- | --- | --- | --- | --- | --- | --- | --- | --- | --- | --- | --- | --- | --- | --- | --- | --- | --- | --- | --- | --- | --- | --- | --- |
|  |
| \|  \| \| --- \| \| \| \| \| **26.** \| **Does your company plan on beginning or increasing the use of rapid RWD analysis in signal assessment over the next 3 years? (Select one)** \| \| --- \| --- \| \| \| --- \| --- \| --- \| \| \|  \|  \| Yes \|  \| \| --- \| --- \| --- \| --- \| \|  \|  \| No - do not believe it will add value \|  \| \|  \|  \| No - there are too many barriers to overcome \|  \| \|  \|  \| Other (Please specify)  __________ \|  \| \| \| \| --- \| --- \| --- \| --- \| --- \| --- \| --- \| --- \| --- \| --- \| --- \| --- \| --- \| --- \| --- \| --- \| --- \| --- \| --- \| --- \| --- \| \| \| \| \| \| **27.** \| **In thinking about how rapid RWD analysis would be used to enhance the quality and speed of signal assessment, what are the most important enablers from your company's perspective? (Free text)** \| \| --- \| --- \| \| \| --- \| --- \| --- \| \|  \| \| \| --- \| --- \| --- \| --- \| --- \| \| \| \| \| \| **28.** \| **Please share any examples where your company found the use of rapid RWD analysis to be impactful. (Free text)** \| \| --- \| --- \| \| \| --- \| --- \| --- \| \|  \| \| \| --- \| --- \| --- \| --- \| --- \| \| \| \| \| \| **29.** \| **If additional information is needed to clarify or expand upon your response, are you willing be contacted by the blinded third party? (Select one) (Select one option)** \| \| --- \| --- \| \| \| --- \| --- \| --- \| \| \|  \|  \| Yes \|  \| \| --- \| --- \| --- \| --- \| \|  \|  \| No \|  \| \| \| \| --- \| --- \| --- \| --- \| --- \| --- \| --- \| --- \| --- \| --- \| --- \| --- \| --- \| \| \| \| \| \| **NOTE :** Answer the below question only if answer to Q#29 is Yes \| \| --- \|      \| **30.** \| **If yes, please provide your email so the blinded 3rd party from TransCelerate can contact you if clarification is needed. (Free text)** \| \| --- \| --- \| \| \| --- \| --- \| --- \| --- \| \|  \| \| \| --- \| --- \| --- \| --- \| --- \| --- \| \| \| \|  \| \| --- \| \| \|  \| |
